# Meaning in Life and Self-Control Buffer Stress in Times of COVID-19: Moderating and Mediating Effects With Regard to Mental Distress

**DOI:** 10.3389/fpsyt.2020.582352

**Published:** 2020-09-23

**Authors:** Tatjana Schnell, Henning Krampe

**Affiliations:** ^1^ Existential Psychology Lab, Institute of Psychology, University of Innsbruck, Innsbruck, Austria; ^2^ Psychology of Religion, MF Norwegian School of Theology, Religion and Society, Oslo, Norway; ^3^ Department of Anesthesiology and Operative Intensive Care Medicine (CCM, CVK), Charité - Universitätsmedizin Berlin, corporate member of Freie Universität Berlin, Humboldt-Universität zu Berlin, and Berlin Institute of Health, Berlin, Germany

**Keywords:** COVID-19, meaning in life, self-control, PHQ-4, crisis of meaning, depression, anxiety, living conditions

## Abstract

**Background:**

As evidenced by several studies, mental distress increased substantially during the COVID-19 pandemic. In this period, citizens were asked to exercise a high degree of self-control with regard to personal and social health behavior. At the same time, we witnessed an increase of prosocial acts and shared creative expressions, which are known to serve as sources of meaning. Meaning in life and self-control are acknowledged psychological resources. Especially in times of crisis, meaning in life has been shown to be a crucial factor for resilience and coping. However, threatening and stressful situations can also jeopardize existential security and trigger crises of meaning. The present study aimed to document levels of acute COVID-19 stress and general mental distress in Germany and Austria during the lockdown and in the weeks thereafter. In order to identify potential risk factors related to demographics and living conditions, their associations with COVID-19 stress were analyzed exploratively. The primary objective of the study, however, was to investigate the buffering effect of two psychological resources—meaningfulness and self-control—with regard to the relation between acute COVID-19 stress and general mental distress. Finally, a potential aggravation of mental distress due to the occurrence of crises of meaning was examined.

**Method:**

A cross-sectional survey was conducted online during lockdown (survey group 1) and the subsequent weeks characterized by eased restrictions (survey group 2). A total of N = 1,538 German-speaking participants completed a questionnaire battery including a novel measure of acute COVID-19 stress, meaningfulness and crisis of meaning (SoMe), self-control (SCS-KD), and a screening of general mental distress, measured by core symptoms of depression and anxiety (PHQ-4). In a first step, associations between living conditions, demographics, and COVID-19 stress were explored. Second, a moderation and a mediation model were tested. Meaningfulness, a measure of presence of meaning in life, as well as self-control were proposed to serve as buffers in a time of crisis, thus moderating the relation between acute COVID-19 stress and general mental distress (double moderation). Crisis of meaning, operationalizing an experienced lack of meaning in life, was proposed to mediate the relationship between acute COVID-19 stress and general mental distress, with an assumed moderation of the association between COVID-19 stress and crisis of meaning by survey group (lockdown versus eased restrictions after lockdown), and a hypothesized moderation of the link between crisis of meaning and general mental distress by self-control (dual moderated mediation).

**Results:**

COVID-19 stress was slightly right-skewed. Scores were higher during lockdown than in the weeks thereafter. The rate of clinically significant general mental distress was high, exceeding prevalence rates from both the general population and clinical samples of the time before the pandemic. In the weeks following the lockdown (group 2), general mental distress and crisis of meaning were significantly higher than during lockdown (group 1), whereas meaningfulness and self-control were significantly lower. Demographically, age had the strongest association with COVID-19 stress, with older participants perceiving less acute stress (r = −.21). People who were partnered or married suffered less from COVID-19 stress (η2 = .01). Living alone (η2 = .006), living in a room versus a flat or house (η2 = .008), and being unemployed due to the pandemic (η2 = .008) were related to higher experience of COVID-19 stress. COVID-19 stress and general mental distress were strongly related (r = .53). Both meaningfulness and self-control were negatively associated with general mental distress (r = −.40 and −.36, respectively). They also moderated the relationship between COVID-19 stress and general mental distress: When meaningfulness was high, high COVID-19 stress was related to substantially lower PHQ-4 scores than when meaningfulness was low. The same held for self-control: High scores of self-control were associated with lower PHQ-4 scores especially when COVID-19 stress was high. Crisis of meaning mediated the relationship between COVID-19 stress and PHQ-4. There was a higher likelihood of crises of meaning occurring when COVID-19 stress was high; crisis of meaning, in turn, was associated with general mental distress. Survey group moderated the first path of this mediation, i.e., the relationship between COVID-19 stress and crisis of meaning: High scores of COVID-19 stress were associated more strongly with crisis of meaning in the second survey group (after the lockdown). Self-control moderated the second path, i.e., the relationship between crisis of meaning and PHQ-4: When a crisis of meaning was present, self-control could buffer its effect on general mental distress.

**Conclusions:**

Also in the present study among German-speaking participants, general mental distress was high. Scores were higher after than during the lockdown, indicating an ongoing destabilization for a significant part of the population. People who saw a meaning in their lives and who were capable of self-control reported substantially less mental distress. Meaningfulness and self-control also served as buffers between COVID-19 stress and general mental distress: When COVID-19 stress was high, the presence of meaningfulness and self-control accounted for lower general mental distress. Moreover, people who suffered strongly from COVID-19 stress were more likely to develop a crisis of meaning which, in turn, was associated with higher general mental distress. This suggests that ongoing anxiety and depression might (also) be based on existential struggles. Again here, self-control buffered the impact of crisis of meaning on general mental health. We conclude from these findings that public health policies can support citizens in coping with large-scale crises by enabling experiences of meaningfulness, e.g., through transparent and reliable modes of communicating goals and necessary intermediate steps. Moreover, health professionals are well advised to invite individuals to confront existential questions and struggles, and to encourage them to exercise self-control. The latter can be boosted by keeping higher-order goals salient—which again is inherently linked to an understanding of their meaning.

## Introduction

Within the first months of the COVID-19 pandemic, evidence from all over the world has accumulated that mental distress has increased substantially. The majority of psychosocial research was conducted in the beginning of the pandemic, when the public atmosphere was dominated by lockdown measures and diverse aspects of uncertainty. From a public health perspective, there was a lack of clarity of how to prevent and reduce most effectively massive waves of outbreaks. From individual perspectives, stress arose from multiple problems associated with the pandemic, such as how to protect oneself and loved ones against infections, confusion, frustration, social isolation, and various fears of the future (1–15).

These stressors took their toll, as indicated by recent studies which report high prevalence rates of clinically significant general mental distress from 16.5% to 46%, of depression from 5.3% to 34.19%, of anxiety from 8.7% to 32.1%, and symptoms of acute stress reactions from 3.8% to 41.8% (8–10, 16–24). A first systematic review and meta-analysis of mental distress during the COVID-19 pandemic found pooled prevalence rates of anxiety, depression, and insomnia of 23.2%, 22.8%, and 38.9%, respectively (25).

These findings suggest that the magnitude of mental health burden caused by the COVID-19 pandemic is comparable to the burden of previous epidemics (26, 27) and of traumatic life events (28). Among the discussed factors to improve mental health associated with epidemics, disasters, and traumatic life events are various treatment approaches, but also public health interventions utilizing resilience factors (4, 26–28). Thus, research on resilience factors and resources that are associated with less mental distress during the recent COVID-19 pandemic will provide important insights for dealing with future crises.

Self-control as well as a sense of meaning in life are acknowledged psychological resources. Especially in times of crisis, meaning in life has been shown to be a crucial factor of resilience and coping ability. However, extremely threatening and stressful situations can also jeopardize existential security and trigger crises of meaning. The present study thus examined a hypothesized buffering effect of meaningfulness and self-control with respect to the relationship between COVID-19 stress and general mental distress, as well as a potential mediation of this relationship by crisis of meaning.

Self-control is defined as the ability to modify or override one’s inner responses as well as to interrupt undesired behaviors (29, 30). It is associated with many indicators of mental well-being, such as satisfaction with life (31), happiness (32), self-esteem (29), and meaning in life (33). Moreover, it is related with lower degrees of depression and anxiety (34). According to longitudinal studies, self-control can predict well-being and health up to 30 years later (35).

Meaning in life is a multi-dimensional construct, covering qualities of experienced meaning in life and sources of meaning (36, 37). Meaning can be experienced as present, i.e., meaningfulness; as absent without ensuing search, i.e., existential indifference; or as painfully lacking, as in a crisis of meaning. In the present study, we will focus on the role of meaningfulness and crisis of meaning during the pandemic. Meaningfulness is the basic trust that life is worth living. It is based on a (mostly unconscious) evaluation of one’s life as coherent, significant, directed and belonging. People with a high sense of meaningfulness are more hopeful and optimistic than people who see little meaning in their lives (38, 39). They experience themselves as more competent, more self-determined, and better socially integrated (40, 41). Their self-regulation abilities are also more pronounced: It is easier for them to activate, motivate, and calm themselves, to direct their attention and to overcome failures (42). They also show higher degrees of self-compassion, self-efficacy, and resilience (33, 43). Meaningfulness is robustly associated with lower mental distress (36, 43–47), higher physical health (48–51), and lower mortality risk (49, 50, 52–54).

A crisis of meaning is defined as a judgement on one’s life as frustratingly empty, pointless, and lacking meaning (36, 37). It is accompanied by disorientation and disintegration of self-view and worldview (55) and is typically associated with depression, anxiety, pessimism and negative mood (36, 38, 43, 45). At the same time, positive affect, life satisfaction, hope and self-efficacy are greatly reduced (36, 38, 45). Also resilience and self-regulation are significantly diminished (42, 43). Crises of meaning were found to predict suicidality among youth independently of depression (56).

A large number of studies have documented that meaningfulness serves as a buffer in times of crisis [see (55, 57)]. It moderates the relationships between stressors and distress, as evidenced for suicide risk factors (58), Alzheimer’s disease (59), traumatic events (60), cancer (47), multiple chronic diseases (61), etc. This buffering effect is reflected in the way individuals deal with and experience stressors, as reported by the above studies: Among those who see meaning in their lives, stressors cause less symptoms and illness behavior, lower perception of pain and suffering, and degrees of mental distress. This suggests that meaning in life may serve as a secure existential foundation that allows people to view stressors more as a worthwhile challenge rather than as harm or loss. Moreover, the purpose fueling a person’s meaning in life can still serve as motivation and compass, even when some pillars of identity break away in times of crisis. A crisis of meaning, on the other hand, often occurs as the result of severe stressors. If not dealt with, it can prevent constructive coping and aggravate distress (62, 63) or provoke self-harming behavior (64).

**Figure 1:**
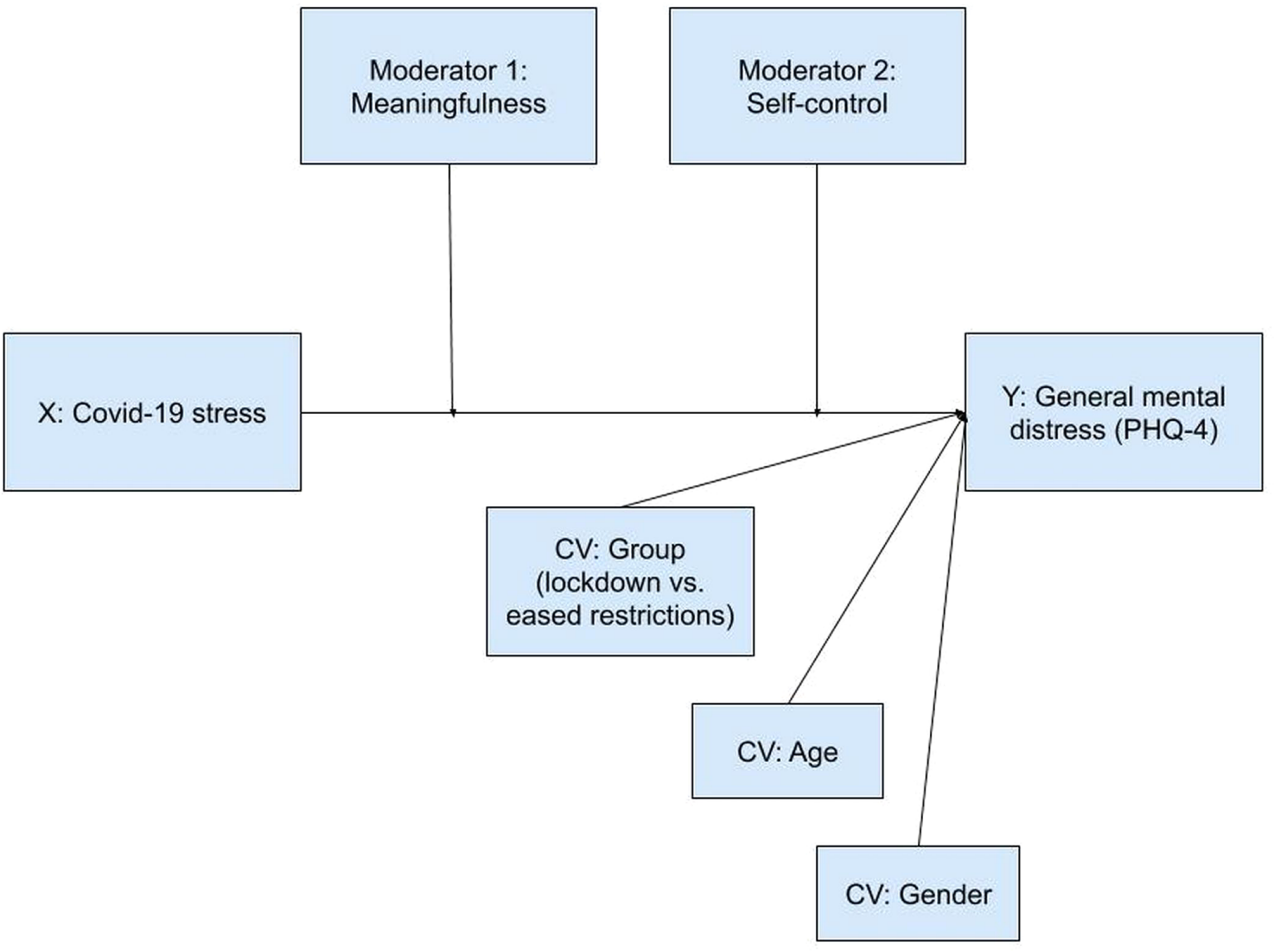
Meaningfulness and self-control moderate the relationship between COVID-19 stress and general mental distress (double moderation, PROCESS model 2).

These findings suggest that people with high degrees of meaningfulness can count on a variety of resilience factors that help them to successfully cope with stressful life events, such as the current pandemic. A high sense of stress due to the pandemic, on the other hand, might jeopardize people’s existential security and bring about a crisis of meaning, which then makes life even harder for them. We thus tested a double moderation and a dual moderated mediation model. First, we expected both meaningfulness and self-control to moderate the relationship between COVID-19 stress and general mental distress (**Figure 1**). Meaningfulness and self-control were hypothesized to serve as buffers with regard to the stress caused by consequences of COVID-19. People who see meaning in their lives and are able to regulate impulses, emotions, and thoughts were expected to be better equipped to deal with restrictions and challenges due to the virus, and thus to be less likely to develop signs of general mental distress.

**Figure 2:**
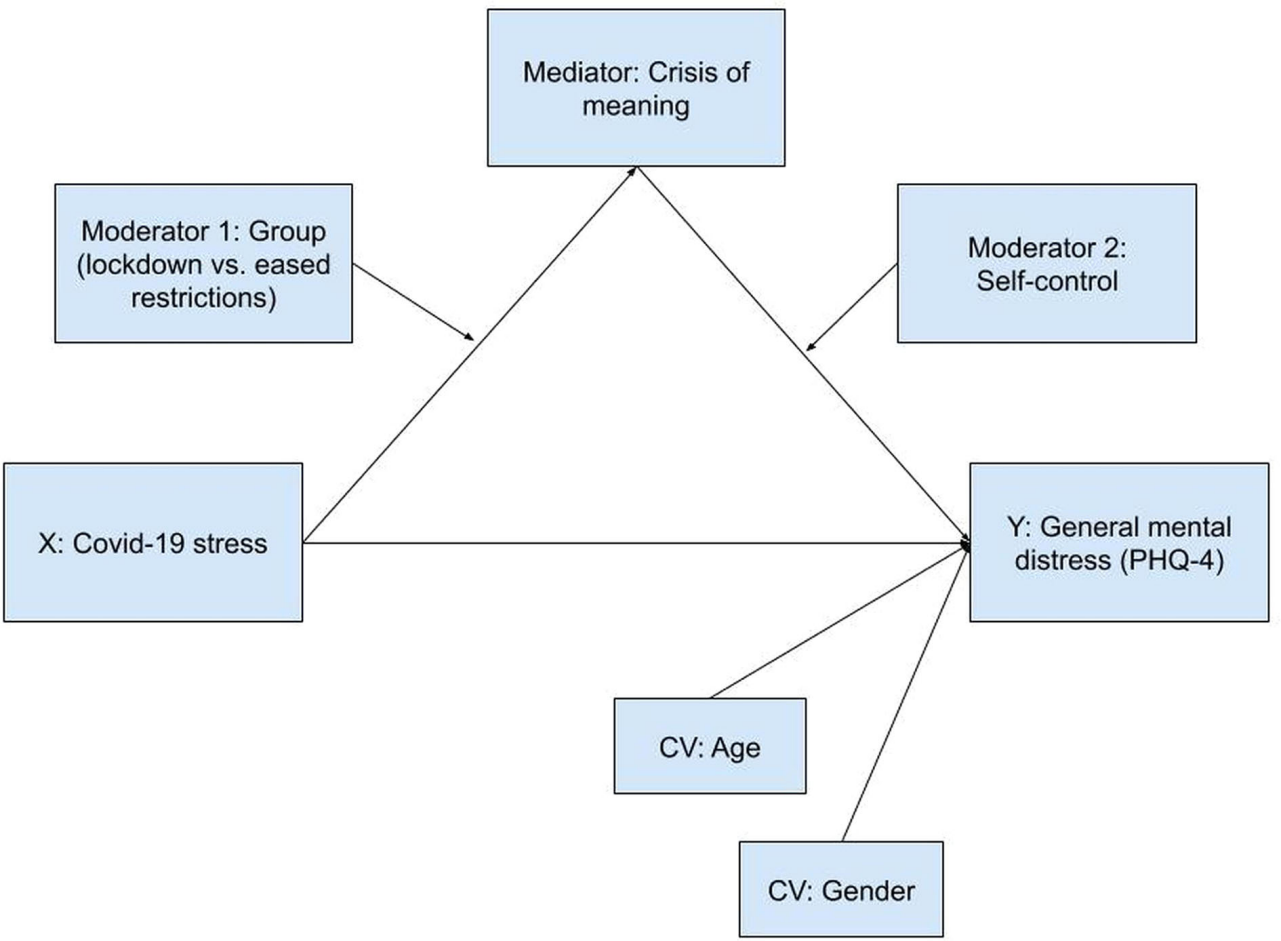
Crisis of meaning mediates the relationship between COVID-19 stress and general mental distress, with self-control and group as moderators (dual moderated mediation, PROCESS model 21).

Moreover, we posited that COVID-19 stress could also result in crisis of meaning [cf. (65)], thus adding to the probability of general mental distress. Also here, we hypothesized that an ability to self-control would attenuate the association between crisis of meaning and general mental distress. We further included survey group (during versus after lockdown) as a moderator of the path between COVID-19 stress and crisis of meaning. This was based on the fact that COVID-19 stress is explicitly situation-related, while crises of meaning are more stable (55) and therefore likely to follow different temporal dynamics. **Figure 2** depicts this model.

## Materials and Method

Using an online questionnaire tool (SoSciSurvey), this cross-sectional survey was carried out in Germany and Austria between April 10 and May 28, 2020. It thus covers three weeks of lockdown and four weeks of increasing ease of restrictions, starting from May 1. Invitations to the study were sent out *via* university, business, and regional network newsletters and posted in several newspapers and news websites. We thus used a kind of convenience sampling with the aim of addressing as many different people as possible. Participation was voluntary, without compensation and could be terminated anytime. Ethical approval was issued by the Review Board (Psychology) of the University of Innsbruck, No 09/2020. All participants expressed their informed consent by explicitly agreeing to continue with the questionnaire after being informed about the study’s aims, employed data protection, participants’ rights and contact points for questions or concerns.

### Participants

A total of N = 1,538 participants completed the questionnaire. For this study, inclusion criteria were a minimum age of 18, agreement to participant consent and completion of the questionnaire. Exclusion criteria were self-report of not having responded honestly (n = 4) and disproportionately short response times (n = 7). After exclusion, a sample of N = 1,527 remained. Of these, 65% (n = 993) identified as women, 35% (n = 528) as men, and 0.4% (n = 6) as divers. (Due to their small number, these were excluded from analyses that contained gender as a variable.) Mean age was 40 (SD = 17; n = 5 missing values), ranging from 18 to 99 years. The majority were German (52%), followed by Austrian (38%). Six percent were of Italian origin, 5% from other nationalities. In terms of highest educational qualifications, 12% had completed their General Certificates of Secondary Education, 30% had an advanced-level qualification, and 58% had a university degree. The majority of participants (99%) had not been diagnosed with COVID-19. The majority also did not know anyone who had been diagnosed (91%) or died of COVID-19 (99%). Fifty-nine percent of the sample participated during lockdown.

### Variables

#### Demographics and Living Conditions

The sociodemographic section assessed participants’ age, gender, nationality, relationship status, children, education, own infection with COVID-19, and infection/death of close persons due to COVID-19. Moreover, we asked participants if they were living alone or with others, about their housing (room, flat or house) and access to a private outside area (balcony, terrace, garden). Finally, the work situation was surveyed (unemployed due to COVID-19 yes/no).

#### COVID-19 Stress

To determine the extent of acute psychological stress due to COVID-19, we developed a novel scale. After examining the relevant literature and drawing on population surveys released by the media, we generated seven items tapping a broad range of affective reactions to the current situation (feelings of intolerability, boredom, anger, and being left alone) and fears and pessimism about internal resources and the future. Items were rated on a six-point Likert scale ranging from 0 (strongly disagree) to 5 (strongly agree). Internal consistency in the present study was Cronbach’s alpha = .71.

#### General Mental Distress

General mental distress was measured by the PHQ-4 (66, 67), a brief four-item measure of core symptoms of current depression and anxiety. It uses a four-point Likert scale ranging from 0 (not at all) to 3 (nearly every day). Participants were asked to respond to the items with a view to the past two weeks. The PHQ-4 has demonstrated good reliability and validity in both clinical and population samples [e.g., (66–70)]. Cronbach’s alpha in this study was .84. Several cut-off points have been validated with ≥3, ≥4, and ≥6 indicating mild, moderate, and severe mental distress (66, 69).

#### Meaning in Life

Two dimensions of meaning in life were assessed by employing the meaningfulness and crisis of meaning scales from the Sources of Meaning and Meaning in Life Questionnaire [SoMe; (36, 71)]. The questionnaire’s reliability and validity have been shown in numerous studies [see (37, 55)]. Meaningfulness measures the degree of experienced meaning in life, and crisis of meaning measures the degree of a perceived lack of meaning. Both five-item scales are rated on a six-point Likert scale ranging from 0 (strongly disagree) to 5 (strongly agree). Internal consistencies in this study were Cronbach’s alpha = .81 and .92, respectively.

#### Self-Control

Self-control was assessed using the validated German version of the SCS [SCS-KD; (29, 72)]. It measures a person’s ability to control their impulses and modify inadequate emotions and thoughts. The 13 items are rated on a five-point Likert scale ranging from 1 (totally disagree) to 5 (totally agree). In the present study, Cronbach’s alpha was .83.

### Statistical Analyses

The primary objective of this study was twofold: to test if two psychological resources—meaningfulness and self-control—would buffer stress resulting from the COVID-19 pandemic by attenuating related general mental distress, and to examine if high levels of COVID-19 stress would be associated with crisis of meaning and, *via* this path, statistically predict elevated general distress. We used PROCESS 3.5 for SPSS (73) to conduct a double moderation (see **Figure 1**) and a dual moderated mediation model (see **Figure 2**). Scale distributions were examined. Skewness and kurtosis were all in acceptable ranges [skewness < 2, kurtosis < 7; cf. (74)]. Parameter estimation used maximum likelihood estimation. Moderation and mediation analyses employed bootstrapping with 5,000 samples. All variables were continuous except for gender (dichotomous: male/female) and survey group (dichotomous: during lockdown/after lockdown). For additional documentation, we report degrees of COVID-19 stress with respect to the temporal context (during/after lockdown), demographics and living conditions. ANCOVAs were used to test for differences.

## Results

### Descriptive Statistics

**Table 1** reports descriptive statistics for all variables during lockdown, immediately afterward, and for the total sample. Additionally, significance levels and effect sizes for differences between the two groups are shown. Since they differed with regard to age, gender, children, and education, these are included as covariates in the comparison between groups (ANCOVAs).

**Table 1:** Means and standard deviations (total), means and standard errors (lockdown and after lockdown), significance levels, effect sizes, and 95% confidence intervals for group comparison.

	Total	Lock- down	After lock- down	*p*	Partial η^2^	*95% CI for the difference*	
						*lower*	*upper*
COVID-19 stress^a)^	1.79 (0.93)	**1.83 (0.03)**	**1.72 (0.04)**	.03	.003	.011	0.215
General mental distress^b)^	3.48 (2.82)	**3.21 (0.10)**	**3.87 (0.12)**	<.001	.01	−.968	−.341
Meaningfulness^a)^	2.94 (1.17)	**3.12 (0.04)**	**2.69 (0.05)**	<.001	.03	.309	.565
Crisis of meaning^a)^	1.16 (1.31)	**0.87 (0.04)**	**1.55 (0.05)**	<.001	.06	−.826	−.539
Self-control^c)^	3.10 (0.69)	**3.14 (0.02)**	**3.01 (0.03)**	.001	.007	.053	.207

N = 1,516; ^a)^range 0–5; ^b)^range 0–12; ^c)^range 1–7; covariates set at age = 40, gender = 1.65 (1-male, 2-female), children (0/1) = 0.36, education = 2.46; bold = significant differences between lockdown and afterward.

**Table 2** shows the prevalence of PHQ-4 scores for different cut-offs discussed in the literature (66, 69), as well as the prevalence of crises of meaning [cut-off according to (75)]. Prevalences are shown in percent, for the total sample, men and women, three age groups, and the two survey groups (lockdown/after lockdown). Chi-square significance levels are given for differences between the two survey groups.

**Table 2:** Percentage beyond cut-off for PHQ-4 and crisis of meaning and significance levels for chi-square test lockdown/after lockdown.

	Total	Gender		Age group			Survey group		*p (chi square)*
		f	m	18–39	40–59	60–99	lock- down	after lock- down	
General mental distress									
% beyond cut-off 3^a)^	56%	60%	50%	63%	51%	44%	58%	54%	.13
% beyond cut-off 4^b)^	41%	44%	35%	47%	37%	31%	42%	40%	.61
% beyond cut-off 6^c)^	19%	20%	17%	22%	18%	10%	**17%**	**22%**	.03
Crisis of meaning									
% beyond cut-off 3^d)^	13%	12%	15%	14%	14%	7%	**9%**	**18%**	<.001

N = 1,521/1,522/1,527; ^a)^at least mild symptoms of depression/anxiety; ^b)^moderate symptoms of depression/anxiety; ^c)^severe symptoms of depression/anxiety; ^d)^presence of a crisis of meaning; bold = significant differences between lockdown and afterward.

Stress due to COVID-19 was right-skewed and thus not wide-spread. It was more marked during lockdown than in the weeks thereafter. Nevertheless, people were apparently affected by the situation, as shown by general mental health scores that surpassed those reported before the virus [e.g., (66–70)]. During lockdown, as many as 58% stated at least mild symptoms of general mental distress, with 42% indicating moderate symptoms and 17% indicating the occurrence of severe symptoms. Suggesting a dynamic different from COVID-19 stress, severe mental distress was more frequent in the second group. After lockdown, both the total score as well as the cut-off score indicating severe symptoms (22%) were significantly higher, while at least mild symptoms were still reported by 54% and moderate symptoms by 40% of the participants. Mirroring this dynamic, also meaningfulness was lower in the second group, compared with levels during lockdown [and also before lockdown, when the average population mean was 3.15 (55)]. Crises of meaning during lockdown appear to have been curbed, with 9% [compared to 14% before the pandemic (55)] reporting scores beyond the cut-off of 3. This also changed after the lockdown, when the number of participants suffering from a crisis of meaning was 18%. For self-control, no reference scores for German speaking populations were available. Mean scores showed a higher level during lockdown than in the immediately following weeks.

Correlations between age and variables included in the moderation and mediation analyses are shown in **Table 3**. COVID-19 stress was highly positively related with PHQ-4. It had moderate negative associations with meaningfulness and self-control, and a moderate to high positive correlation with crisis of meaning. COVID-19 stress, PHQ-4, and crisis of meaning were less pronounced among older participants; these also had slightly higher scores in self-control and meaningfulness.

**Table 3:** Correlations between study variables and age.

	COVID-19 stress	PHQ-4	Age
COVID-19 stress			−.21
Meaningfulness	−.28	−.40	.13
Crisis of meaning	.41	.65	−.12
Self-control	−.21	−.36	.17
PHQ-4	.53		−.17

N = 1,522; Pearson correlation; all coefficients significant at p <.001.

### Predicting COVID-19 Stress

Before testing our main hypotheses, we report potential predictors of increased stress due to COVID-19. **Table 4** shows associations between living conditions, demographics, and COVID-19 stress, controlling for survey group. The biggest effect can be attributed to age, with older participants experiencing significantly less stress due to COVID-19. Due to this finding, age was controlled in all other analyses. According to the data, people who were married or partnered experienced less stress. Participants of Italian origin reported higher stress than all others. So did individuals who were unemployed due to COVID-19, and who lived alone or in a room instead of a flat or house.

**Table 4:** Living conditions and demographics affecting COVID-19 stress: estimated means, standard errors, significance levels, effect sizes, and 95% CI.

		*n*	COVID-19 stress		*p*	Partial η^2^	*95% CI for est. M*	
			Est. M	SE			*LL*	*UL*
**Age** ^a)^					<.001	.03		
	18–39	817	**1.93**	(0.03)			1.87	2.00
	40–59	462	**1.65**	(0.04)			1.56	1.73
	60–99	243	**1.57**	(0.06)			1.45	1.68
Gender^b)^					.08	.002		
	Female	993	1.81	(0.03)			1.76	1.87
	Male	528	1.73	(0.04)			1.65	1.80
**Nationality** ^b)^					.004	.009		
	German	792	**1.80**	(0.04)			1.73	1.87
	Austrian	573	**1.71**	(0.04)			1.63	1.79
	Italian	89	**2.08**	(0.10)			1.89	2.28
	Other	73	**1.79**	(0.11)			1.58	2.00
**Relationship status** ^b)^					<.001	.01		
	Married/partnered	953	**1.71**	(0.03)			1.99	2.24
	Other	574	**1.90**	(0.04)			1.84	2.11
Children^b)^					.74	.00		
	Yes	552	1.77	(0.05)			1.68	1.86
	No	975	1.79	(0.03)			1.73	1.85
Education^b)^					.36	.001		
	Secondary	190	1.84	(0.07)			1.71	1.98
	Advanced level	453	1.81	(0.05)			1.73	1.90
	University	884	1.76	(0.03)			1.70	1.82
**Living alone** ^b)^					.002	.006		
	Alone	328	**1.92**	(0.05)			1.82	2.02
	With others	1,199	**1.75**	(0.03)			1.69	1.80
**Housing** ^b)^					.001	.008		
	Room	134	**2.04**	(0.08)		1.89	2.20	
	Flat or house	1,393	**1.76**	(0.02)			1.71	1.81
Access to outside^b)^					.09	.002		
	No	216	1.88	(0.06)			1.76	2.01
	Yes	1,311	1.77	(0.03)			1.72	1.82
**Work situation**					.001	.008		
	Unemployed due to COVID-19	72	**2.14**	(0.11)			1.93	2.35
	Other	1,455	**1.77**	(0.02)			1.72	1.81

^a)^ANCOVA controlling for group (1/2; set at 1.41); N = 1,527; ^b)^ANCOVA, controlling for age (set at 40) and group (set at 1.41); N = 1,522. CI, confidence interval; LL, lower limit; UL, upper limit; bold, significant differences.

### Meaningfulness and Self-Control Moderate Effects of COVID-19 Stress on General Mental Distress

To test the hypothesis that general mental distress is a function of personal characteristics and external stressors, and more specifically whether meaningfulness and self-control would moderate the relationship between COVID-19 stress and general mental distress, a double moderation analysis was conducted (using PROCESS 3.5 macro for SPSS, model 2). Because values differed significantly between lockdown and the time thereafter, group was included as a covariate. Because COVID-19 stress was related to age and PHQ-4 scores are known to be related to gender (68–70), also these two variables were included as covariates. All variables defining products were mean centered.

The model was significant at F (8, 1507) = 122.8789, p <.001, R^2^ = .40. COVID-19 stress, meaningfulness, self-control, survey group and gender explained 40% of variance in general mental distress. Both meaningfulness and self-control acted as independent moderators of the association between COVID-19 stress and general mental distress, as shown by statistically significant interactions (see **Table 5**). Addition of the interaction between meaningfulness and COVID-19 stress yielded an F(1, 1507) = 11.17, p = .001, change R^2^ = .005; addition of the interaction between self-control and COVID-19 stress F(1, 1507) = 4.49, p = .03, change R^2^ = .002. The inclusion of both interactions yielded an F(2, 1507) = 11.65, p <.001, change R^2^ = .009.

**Table 5:** Double moderation of COVID-19 stress predicting general mental distress.

Effect	Estimate	*SE*	*t*	*95% CI for estimate*		*p*
				*LL*	*UL*	
Intercept	2.38	0.32	7.40	1.74	2.99	<.001
COVID-19 stress (IV)	1.27	0.07	19.21	1.14	1.40	<.001
Meaningfulness (Mod 1)	−0.50	0.05	−9.16	−0.60	−0.39	<.001
Interaction IVxMod 1	−0.18	0.05	−3.22	−0.27	−0.07	.001
Self-control (Mod 2)	−0.81	0.09	−9.25	−0.98	−0.64	<.001
Interaction IVxMod 2	−0.18	0.09	−2.24	−0.36	−0.02	.03
Group^a)^	0.44	0.13	3.36	0.18	0.69	.001
Gender^b)^	0.38	0.15	3.01	0.13	0.62	.003
Age	−0.01	.00	−1.31	−0.01	−0.00	.19

N = 1,516. CI, confidence interval; LL, lower limit; UL, upper limit. ^a)^lockdown = 1, after lockdown = 2.^b)^male = 1, female = 2.

**Figure 3:**
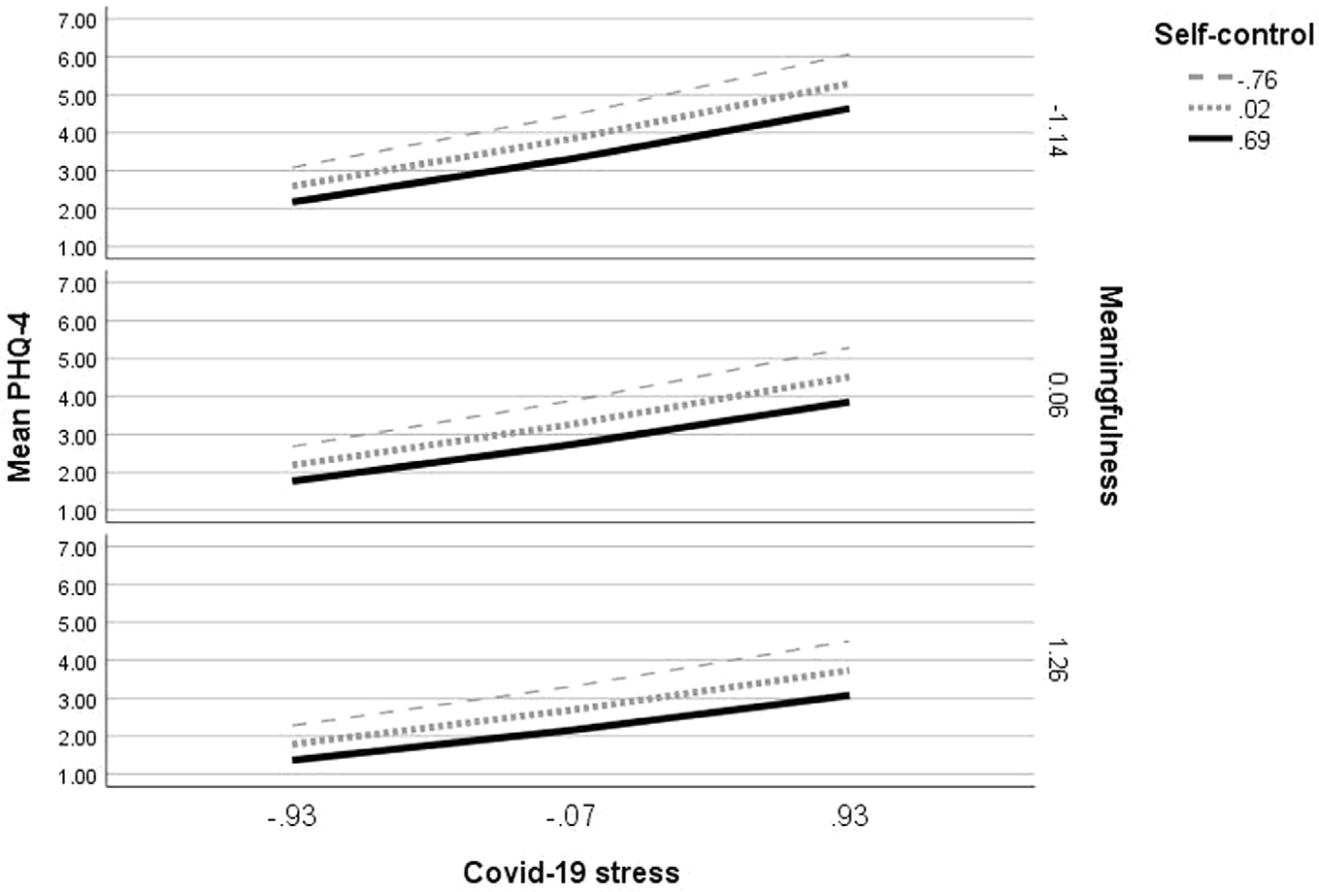
Meaningfulness and self-control moderating the relationship between COVID-19 stress and general mental distress.

As an examination of the interaction plots shows (see **Figure 3**), general mental distress increased with COVID-19 stress. The increase was attenuated by both meaningfulness and self-control. At differing degrees of COVID-19 stress, general mental distress decreased when meaningfulness increased (lower PHQ-4 levels in the second and third row). General mental distress also decreased when self-control increased (higher PHQ-4 levels in the dashed and dotted lines). Participants with low meaningfulness and low self-control had the highest PHQ-4 scores. These ranged from average PHQ-4 scores of around 3 when COVID-19 stress was low, to average PHQ-4 scores of around 6 when psychological strain due to COVID-19 was high. Effects of both meaningfulness and self-control specifically showed when COVID-19 stress was high (right hand side of the figure).

### Crisis of Meaning as a Moderated Mediator Between COVID-19 Stress and General Mental Distress

Our second hypothesis suggested that increased suffering due to COVID-19 might jeopardize existential security and thus instigate crises of meaning. These, in turn, have been shown to further increase mental suffering (62, 63). This hypothesis frames crisis of meaning as a mediator between COVID-19 stress and general mental distress. Also here, we assumed that perceived self-control would reduce the probability of experiencing general mental distress. Self-control should thus moderate the path between crisis of meaning and PHQ-4. Moreover, we included another moderator: As could be seen in the preliminary analysis testing for differences between lockdown and the weeks thereafter, COVID-19 stress was lower in the second group, but crisis of meaning was higher. We therefore posited that group would moderate the path between COVID-19 stress and crisis of meaning. Again, age and gender were included as covariates. **Table 6** displays the results of the dual moderated mediation (using PROCESS 3.5 macro for SPSS, model 21). All variables defining products were mean centered.

**Table 6:** Dual moderated mediation.

Effect	Crisis of meaning (Mediator)						General mental distress (DV)					
	Est.	*SE*	*t*	*95% CI for est.*		*p*	Est.	*SE*	*t*	*95% CI for est.*		*p*
				*LL*	*UL*					*LL*	*UL*	
Intercept	.82	.14	5.69	0.53	1.10	<.001	2.91	.25	11.61	2.41	3.41	<.001
COVID-19 stress (IV)	.61	.03	18.74	0.54	0.67	<.001	.91	.06	14.89	0.79	1.03	<.001
Crisis of meaning (Med)							1.00	.05	21.29	0.90	1.09	<.001
Group (Mod 1)	.77	.07	11.64	0.63	0.90	<.001						
Interaction IVxMod 1	.31	.07	4.66	0.18	0.44	<.001						
Age	−.01	.00	−6.49	−0.02	−0.01	<.001	−.00	.00	−0.56	−0.01	0.01	.58
Gender^a)^	−.17	.06	−2.65	−0.29	−0.04	.008	.35	.11	3.20	0.14	0.57	.012
Self-control (Mod 2)							−.44	.08	−5.54	−0.60	−0.29	<.001
Interaction MedxMod 2							−.17	.05	−3.10	−0.27	−0.06	.001
	R^2^ = .25 F(5,1510) = 102.3614, p <.001						R^2^ = .52 F(6,1509) = 269.1002, p <.001					

N = 1,516. CI, confidence interval; LL, lower limit CI; UL, upper limit CI. ^a)^ male = 1, female = 2.

**Figure 4:**
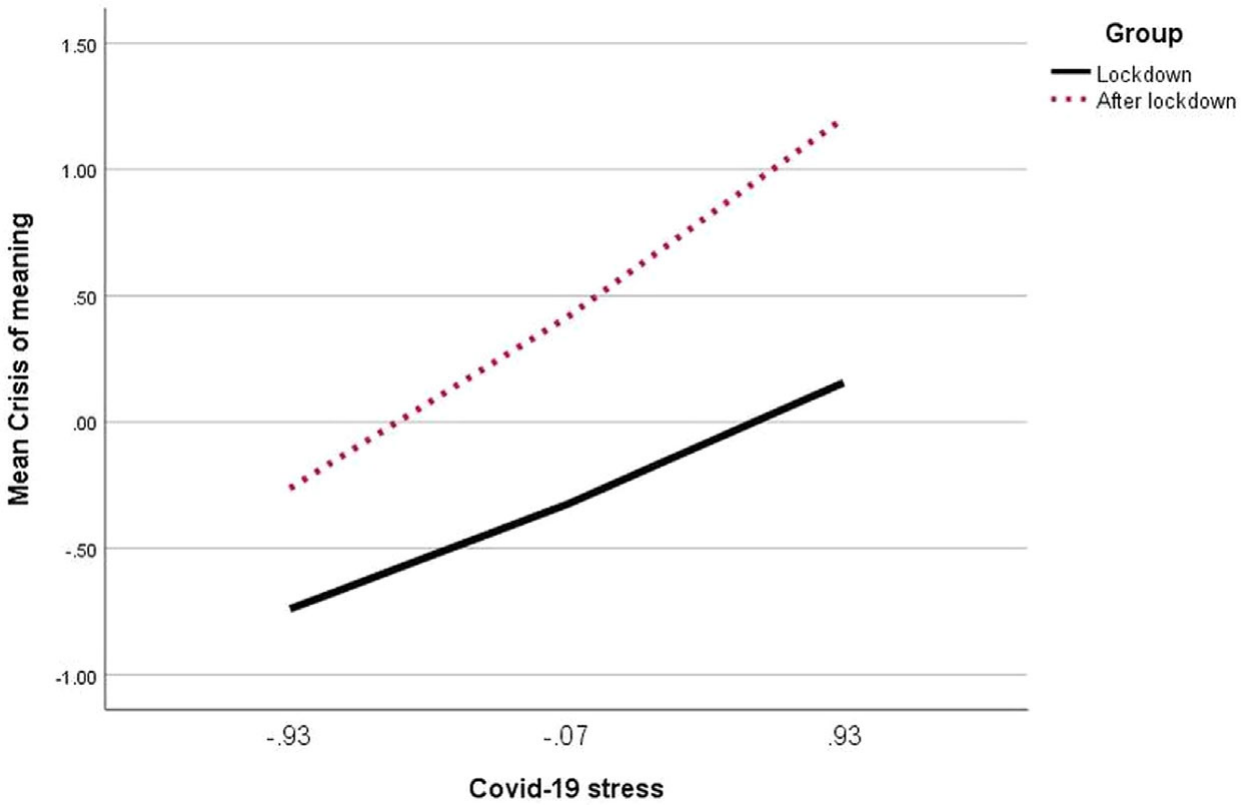
Survey group moderating the relationship between COVID-19 stress and crisis of meaning.

COVID-19 stress, group, and an interaction of both explained 25% of variance in crisis of meaning (see **Table 6**), with crisis of meaning being higher when people experienced stress due to COVID-19, and after lockdown. The interaction between COVID-19 stress and group was also significant, indicating that when suffering due to COVID-19 was still high after lockdown, crisis of meaning was especially prominent (see **Figure 4**). Addition of the interaction between COVID-19 stress and group yielded an F(1,1510) = 21.72, p <.001, change R^2^ = .01.

**Figure 5:**
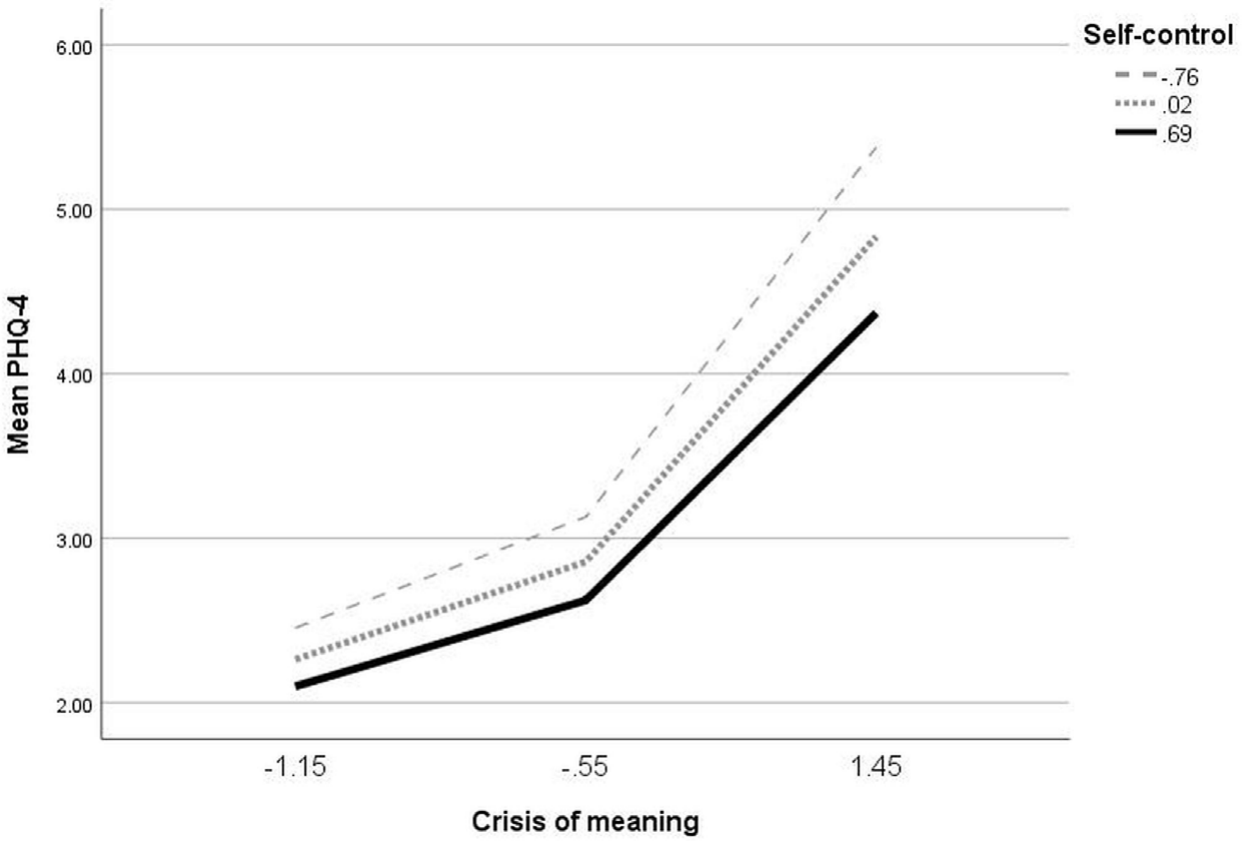
Self-control moderating the relationship between crisis of meaning and general mental distress.

COVID-19 stress and crisis of meaning were also positive predictors of general mental distress, and self-control predicted it negatively. With 52%, a substantial amount of variance in general mental distress could be explained by the predictors and their interactions. The data showed the expected interaction between crisis of meaning and self-control, as illustrated by the plotted interaction in **Figure 5**. The higher crisis of meaning, the larger was the effect of self-control with regard to general mental distress. Especially when crisis of meaning was high, people with high self-control suffered significantly less from general mental distress than people with low self-control. Addition of the interaction between crisis of meaning and self-control yielded an F(1,1509) = 9.61, p = .002, change R^2^ = .003.

The index of the moderated mediation was −.05 (SE = .02, 95% CI −0.10/−0.01), thus supporting the hypothesis that the indirect effect was conditional on the level of the moderator variables: Group and self-control significantly moderated the indirect effect of crisis of meaning on general mental distress. All indices of conditional moderated mediation by group among low, medium, and high degrees of self-control were significant, indicating that the moderation of the indirect effect by group differed with varying degrees of self-control. (Indices and estimates for the indirect effects are available from the first author upon request.)

Due to the cross-sectional character of the present study, causal effects cannot be determined. To test alternative directions in the present model, we also carried out the above analysis with the following models: crisis of meaning as independent variable and COVID-19 stress as mediator (index of moderated mediation = .003, SE = .01, 95% CI −0.01/0.02). Crisis of meaning as independent variable and PHQ-4 as mediator (index of moderated mediation = .001, SE = .00, 95% CI −0.01/0.01). PHQ-4 as independent variable and COVID-19 stress as mediator (index of moderated mediation = .005, SE = .00, 95% CI 0.00/0.01). PHQ-4 as independent variable and crisis of meaning as mediator (index of moderated mediation = .002, SE = .00, 95% CI −0.00/0.01). Since all 95% CIs included zero, none of these indices was significant, thus supporting the hypothesized model.

## Discussion

The present study examined degrees of acute psychological stress due to COVID-19 and general mental distress reported by German-speaking participants during the lockdown (survey group 1) and in the subsequent weeks (survey group 2) of the COVID-19 pandemic.

### COVID-19 Stress Not Widespread, But Related to Mental Distress

On average, COVID-19 stress was moderately marked. Most participants only felt low to moderate degrees of intolerability, boredom, anger, or being left alone; their view of the future was neither very fearful nor pessimistic. After restrictions had been eased, stress due to COVID-19 was lower, too. However, those participants who did report higher stress due to COVID-19 also expressed elevated general mental distress. This has also been reported by studies from other countries, such as Bangladesh (13), Canada (10), Iran (1), Israel (3), Italy (76), Turkey (15), and the United States of America (6, 7, 10).

### Demographics and Living Conditions as Predictors of COVID-19 Stress

The emerging picture portraits German and Austrian citizens as largely unaffected by the crisis [see also (77)]. For a smaller, but not unsubstantial part of society, however, longer-term mental health problems seem to be emerging. According to our analysis of demographic parameters and living conditions, these were only fragmentarily responsible for the reported strain. We saw higher acute stress scores among younger people, people who had no partner, who lived alone and in constricted housing conditions. Unemployment due to the pandemic also predicted higher COVID-19 stress. In more heterogeneous populations, these effects might be larger than the ones found here, since the present sample was marked by relatively high education and comfortable living conditions. Nevertheless, these findings add to practical knowledge about predictors of critical experiences by specific subgroups during large-scale events (1, 3, 5–7, 9–13, 15, 76), especially since recent studies have scarcely reported results on associations between COVID-19 stress and demographic factors and living conditions.

Concerning age, Lee reported a significant negative correlation with the Coronavirus Anxiety Scale (CAS) total score of r = −0.32 (6), which is comparable to, but slightly larger than the negative correlation in the study at hand (r = −0.21). Other authors found no relations between age and their respective measures of COVID-19 stress (5, 6, 11, 13, 76). Among the investigations of gender and COVID-19 stress, two studies also reported no differences between men and women (6, 11), while four found higher stress in women than in men (3, 5, 13, 78).

### Meaningfulness and Self-Control Serve as Stress Buffers

Besides investigating the occurrence of mental and existential distress during the pandemic, the present study posited positive direct and moderating effects of two psychological resources, i.e., meaningfulness and self-control. As expected, acute COVID-19 stress was associated with substantially less general mental distress when people saw a meaning in their lives, and when they perceived themselves as capable of exercising self-control. The proposed buffering effect of meaning in life was thus supported, suggesting that a sense of meaningfulness provides a stable existential foundation to cope with critical life-events. The data also indicated that self-control served as a buffer of acute suffering due to COVID-19, since this strain was associated with lower general mental distress when self-control was high.

Both resources seem to have supported a lot of people during the lockdown. Concerningly, both were less marked in the data collected after the lockdown. The difference was particularly marked for meaningfulness, which changed from M = 3.12 to M = 2.69, a score much beyond the average seen in the years preceding the pandemic (55). This finding must be interpreted with caution, since the participants after the lockdown were not the same as before, and we can therefore not talk about personal changes. Nevertheless, and considering that demographics were controlled, the systematic differences between the two survey groups suggest some association with the time of the survey. Lower meaning scores after lockdown mirror the high level of severe mental distress, but they also point beyond personal levels of suffering. Perceptions of meaning are based on the evaluation of immediate circumstances with reference to higher-level contexts (36, 37), implying a so-called “surplus of meaning” [(55), p. 28ff]. Therefore, acts are perceived as meaningful when they result in intended goals, and goals are perceived as meaningful when they concur with higher order life purpose/sources of meaning. During the COVID-19 pandemic, citizens were required to act in different-than-usual ways. Courses of action were drastically restricted. Inferring from this study’s data, the majority of participants could see their lives and actions as meaningful in spite of these restrictions—perhaps due to clear and consistent communication of government policies. During the lockdown, Austrian and German governments clearly communicated which kinds of action were demanded to pursue also quite clearly communicated goals. These goals were further justified by higher order objectives, i.e., putting health concerns first and safeguarding those who are weak and at risk. Moreover, the policies were brought into force in a way that enhanced all four facets contributing to a sense of meaning: Each person mattered (significance); the direction of action was clear and justified (orientation); directives applied to everybody and all areas of life (coherence); everybody was at risk and the only reasonable reaction was to be a collective effort (belonging).

### Insecurity After the Lockdown

Experiences seem to have changed considerably after restrictions were started to be eased. The “exit” was characterized by a much higher degree of insecurity, by inconsistencies and contradictions in the communication of guidelines and by different strategies employed by different regions. Under such conditions, the meaningfulness of one’s actions is much less apparent and the perceived meaningfulness of prescribed goals is easily jeopardized by ambiguous rationales and communication. This situation is the context in which we found that acute COVID-19 stress was lower compared to the time of lockdown, while PHQ-4 scores were even higher than during the lockdown. Negative psychological reactions to the pandemic thus either take place time-delayed or derive from a state of insecurity and incoherence rather than from clearly communicated restrictions in social behavior. This also showed in crisis of meaning scores. In the weeks following the lockdown, crises of meaning were twice as frequent as during the lockdown. This increase could be attributed to various causes. On the one hand, it could be due to material existential worries that were not identified in this study. On the other hand, it is possible that individuals were motivated by the external restrictions to a more intense reflection of their inner lives, their goals and beliefs. From psychotherapy and posttraumatic growth research we know that in situations of crisis a reorientation often occurs, which in many cases is preceded by the abandonment or destruction of the previous life plan or even worldview (79). Thus, crises of meaning could be understood as a transitional phase to a more realistic life- and worldview. Last but not least, increases in crises of meaning could be related to the state of society after the lockdown, as characterized above. In addition to the prevalent insecurity, the reinstatement of a “new normality” is prone to belie expectations of fundamental changes in social, ecological, and economical matters as held and voiced by many during the pandemic [see, e.g., (80)].

### Long-Term Ego Depletion Effects?

At the same time, and perhaps connected with these developments, we saw lower scores of perceived self-control in the second survey group of our study. Also this finding must be interpreted with caution; because the study design was not longitudinal, we cannot speak of a decrease in self-control. However, lower scores after lockdown tie in with evidence for a spreading ignorance of social distancing in society [e.g., (81)] and numerous protests against further coronavirus restrictions (82). For explanation of this phenomenon, ego depletion theory might be considered, i.e., an impairment of subsequent self-control after initial exertion of self-control. This is also known to pertain to moral behavior, which becomes less likely after initial exertion of self-control (83). The effect has so far been associated with much shorter time frames, typically in experimental settings. The original explanation of ego depletion—the strength model (84, 85)—assumed that exertion of self-control draws on a limited pool of mental resources and can thus be “used up”. This understanding has been contested [for a meta-analysis, see (86)]. Alternatively, ego depletion effects are attributed to reduced motivation to engage in further self-control (87, 88). Studies that reduced ego depletion by priming goals (89) or self-awareness (90) support this explanation.

These studies also suggest an inherent connection between self-control and meaningfulness. Top-down control processes that modulate or inhibit predominant responses can be boosted by reminding people of the reasons for exercising self-control, i.e., their higher order goals (91). According to the hierarchical model of meaning (37, 55), meaningfulness is based on coherence between action, goals, and higher order purpose/sources of meaning. When public health policies are communicated in a way that ties in with life orientations held by a majority of the society and the link between these values and particular goals is clear and comprehensible, then citizens have the chance to identify with these values, adopt the respective goals and orient their behavior accordingly, including the exercise of self-control, when necessary.

### Limitations

The study at hand used a large, but cross-sectional sample in order to yield early insights into German-speaking participants’ mental health and existential standpoints. Therefore, a direction of effects as implied by mediation models cannot be determined. We tried to mitigate this problem by testing models with different implied directions. All of these did not yield significant results, thus suggesting that they are less probable than the originally hypothesized and supported model. Follow-up studies are programmed and the findings reported here will be replicated in longitudinal designs.

Our main outcome measure, the PHQ-4, does not establish diagnoses of depression or anxiety according to ICD-10 or DSM-5. It measures core symptoms of both, thus indicating, by means of several cut-off scores, occurrence of at least mild, moderate, or severe, clinically relevant symptoms. The PHQ-4 has been demonstrated to be a valid screening tool for general mental distress in the general population and clinical populations [e.g., (66–70)].

The COVID-19 stress scale was newly developed for the current investigation, as no validated instruments were available at the time we initiated the study. Preliminary indications of its validity can be inferred from the fact that the scale correlated with demographic characteristics and indicators of mental distress in a comparable way to published validated scales (1, 3, 5–7, 10–13, 15, 76). Also, its relationships with other constructs in our study corresponded to our hypotheses and can thus be considered as first evidence for construct validity.

Finally, the sampling we used is prone to several limitations. As in the majority of studies, it is impossible to determine why some people chose to take part and others did not. The results can therefore not be generalized to the population as a whole. We also cannot determine the response rate since we do not know how many subjects read the open invitation to participate in the study.

### Conclusions and Implications

This study indicated that younger people might be more vulnerable than older to suffer from stress due to COVID-19. Stress was also related to living alone and in confined housing conditions, and to unemployment due to the pandemic. These population groups should thus be given special attention in large-scale crises. We saw concerningly high general mental distress and crises of meaning especially in the time after the lockdown, suggesting that long-term negative developments were triggered by the lockdown or the handling of the exit. Mental health support services should be made widely accessible to prevent psychological suffering. With regard to crises of meaning, it should be noted that they are not exclusively negative, but also hold potential for personal growth (75, 92, 93). Especially here, therefore, care should be taken not only to eliminate the symptoms of suffering, but to take arising questions seriously and search for possibilities of fundamental change and improvement. This also applies to crises at social level. Many initiatives and citizens’ movements currently share their visions and proposals for a better future (e.g., #EUvsVirus Hackathon). A great opportunity lies in involving them in social development processes in a participatory manner, and to listen to voices in all their diversity. This might be a way of preventing a growing number of people from experiencing themselves as alienated and powerless, with the possible effect of turning to conspiracy theories [cf. (94)].

Including diverse groups in decision and policy making processes can also enable citizens’ sense of meaning, which our data showed to work as a buffer in critical times: people who saw a meaning in their lives were less affected by acute COVID-19 stress and by general mental distress; when their COVID-19 stress was high, it was associated with significantly lower general mental distress. Experiences of meaning are based on four facets, i.e., *significance, coherence, orientation*, and *belonging* (37, 55). All four facets can be strengthened by the implementation of democratic values: Significance is the experience of mattering, of making a difference. Mattering is enhanced by being heard and seen, by being offered real possibilities of participation and attribution of responsibility [e.g., (95)]. Coherence is based on comprehensibility and consistency, which are mutually dependent: The more we understand about ourselves and our world, the better we can orientate our actions accordingly, thus creating coherence and consistency. In practical terms, it should be ensured that sufficient information also reaches those sections of the population who, for linguistic or infrastructural reasons, have no access to the usual channels. This might be the case for, e.g., migrant workers with little knowledge of German. Orientation refers to the direction pursued. The more clearly communicated and justified it is, the easier it is for citizens to position themselves. Here, it is of importance for governments to elaborate the societal norms and values it bases its decisions on, and how specific codes of practice—such as physical distancing and personal hygiene—as well as measures like economic lockdown concur with these values. Honesty and transparency in this regard will again affect coherence. Fourth, a sense of belonging can strengthen joint action when needed, and counter-act a disintegration of society. The more citizens perceive themselves as part of society, or humanity, the more they will be willing to act responsibly. This should be kept in mind especially with regard to groups of people who consider themselves marginalized, and insignificant.

Finally, our data confirmed the importance of self-control as a buffer attenuating the link between COVID-19 stress and general mental distress. Being a top-down process, self-control is most likely when we know why we *should* modify or interrupt our desires. Again, modes of communicating governmental policies play a major role here. Some governments may choose to communicate in a way that induces fear, as has apparently been the case in Austria (96). Indeed, fear of COVID-19—also termed “functional fear”—has been established as a stable predictor of compliant behavior change (97). But, there are alternatives to this. When policy making is based on multi-perspective advice (covering, e.g., medical and social science, economy and philosophy), when policy communication is clear and substantiated, when citizens are invited to express questions and objections as, e.g., in round-table meetings or other forms of democratic participation [cf. (82)], then self-control is not obedience, but a possible outcome of informed personal decision.

## Data Availability Statement

The raw data supporting the conclusions of this article will be made available by the authors, without undue reservation.

## Ethics Statement

The studies involving human participants were reviewed and approved by the Review Board (Psychology) of the University of Innsbruck. Written informed consent for participation was not required for this study in accordance with the national legislation and the institutional requirements.

## Author Contributions

TS conceptualized the project and analyzed the data. TS and HK collected and interpreted the data, drafted and revised the manuscript, and read and approved the submitted version of the manuscript.

## Conflict of Interest

The authors declare that the research was conducted in the absence of any commercial or financial relationships that could be construed as a potential conflict of interest.
